# From the CONSORT to the ConPhyMP statement and beyond—how to ascertain best practice

**DOI:** 10.3389/fphar.2023.1338710

**Published:** 2023-12-11

**Authors:** Michael Heinrich, Banaz Jalil

**Affiliations:** ^1^ Research Group Pharmacognosy and Phytotherapy, UCL School of Pharmacy, University London, London, United Kingdom; ^2^ Chinese Medicine Research Center, Department of Pharmaceutical Sciences and Chinese Medicine Resources, College of Chinese Medicine, China Medical University, Taichung City, Taiwan

**Keywords:** Chemical analysis, traditional medicine, phytotherapy, HPTLC, HPLC, *in vitro* pharmacodynamics, clinical trial, ConPhyMP

## Abstract

With the implementation of the ConPhyMP reporting tool as an element of peer review in Frontiers in Pharmacology, Section Ethnopharmacology and in other journals, this short perspective paper highlights the use of a new tool available via the website of the Society for Medicinal Plant and Natural Product Research (https://ga-online.org/best-practice/) and how to use it. The ConPhyMP guidelines and the tool cover the relevant aspects which need to be reported when studying a plant extract using pharmacological, toxicological microbiological, clinical and other approaches. In our vision, science will only remain impactful if it is based on a drive for best practice, i.e., on a sound conceptual and methodological basis.

## Introduction

Transparency in reporting and the wider reproducibility of the research outcomes are one of the most fundamental aspects of any research project and was, for example, the driving force behind the CONSORT statement (http://www.consort-statement.org/Consolidated Standards of Reporting Trials), which was first developed 40 years ago in 1993 during a meeting in Ottawa, Canada and formally published as a draft in 1994 and in its full version in 1996 (Standards of Reporting Trials Group, 1994; Begg et al., 1996). We are facing a crisis of a lack of reproducibility relevant to all fields of pharmacology, toxicology, clinical research and related fields. As a consequence, research cannot be used to make evidence-based decisions and to improve healthcare practice. Specifically, in the case of research on extracts, this is exacerbated by unclear, incomplete or simply incorrect descriptions of the material studied, a problem we face in all journals in this field of research. Our vision with this is to make science more transparent, reproducible, and, therefore, impactful.

## The ConPhyMP statement and the associated online tool

An initiative used a three-stage process of community consultation, a Delphi process, and lastly, the gathering of feedback by key expert stakeholders on the Consensus-based reporting guidelines for the Phytochemical Characterisation of Medicinal Plant extracts (ConPhyMP) (Heinrich et al., 2022) were achieved, and it is a part of the requirements when submitting manuscripts to Frontiers in Pharmacology, Section Ethnopharmacology and is being implemented in other journals. The focus is on medicinal plant extracts used in pharmacological, toxicological, and clinical/intervention research to ensure the reproducibility of research methodology and outcomes, hence, the accurate interpretations of studies using medicinal plant extracts.

**FIGURE 1:**
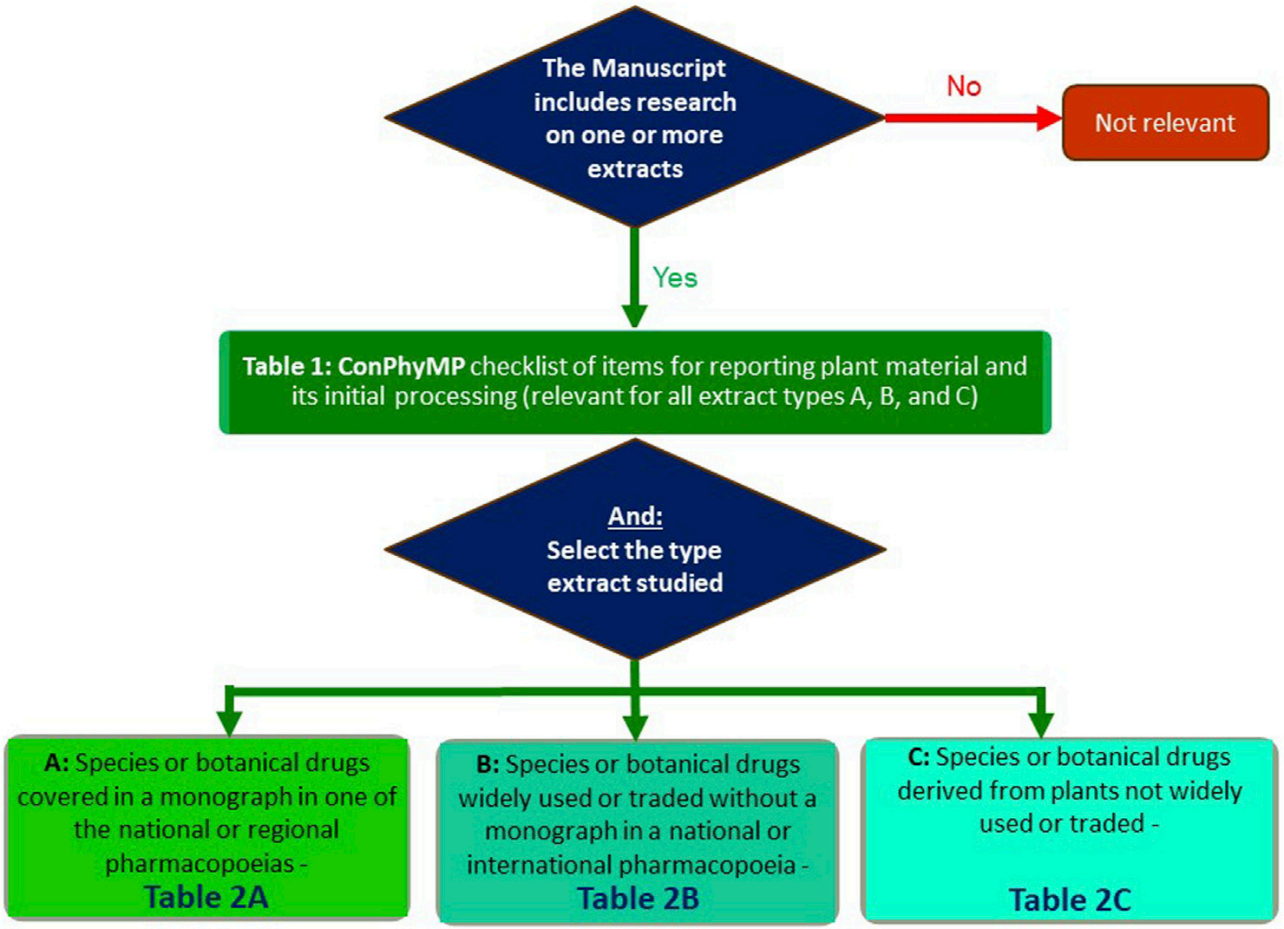
Flowchart for the use the ConPhyMP guidelines (Heinrich et al., 2022) and ConPhyMP open access tool (https://ga-online.org/best-practice/).

In order to facilitate its use, an open-access tool is now available: https://ga-online.org/best-practice/. It is hosted by the Society for Medicinal Plant and Natural Product Research (GA). It covers the relevant aspects which need to be reported when studying a plant extract. In order to use it, you will need to complete two tables/checklists–Table 1 and one of the three options for Table 2 (A, B, or C) of the tool (Figure 1):

Table 1 of the tool (ConPhyMP checklist of information for reporting plant material and its initial processing) is relevant for all extract types and covers the basic requirements, including details about the species and botanical drug used, its source, initial processing extraction, and, if applicable, formulation (Heinrich et al., 2022). It is needed for all manuscripts, and we expect it to be submitted as a part of the Supporting Material. If you are studying a metabolite isolated from a plant/fungus/algae/etc., it is important to assess your manuscript against Table 1 of the tool (Figure 1).

In the case of Table 2 of the tool, three options exist, and these are based on the relevance of the botanical extract as a medicine, food supplement or any other use where pharmacological, toxicological, food functional or clinical data are to be reported (Heinrich et al., 2022):

• Extract type A (for species or botanical drugs covered in a monograph in one of the national or regional pharmacopoeias).
• Extract type B (for species or botanical drugs widely used or traded without a monograph in a national or international pharmacopoeia)
• Extract type C (for species or botanical drugs derived from plants not widely used or traded)

Taking Table 2A of the tool (Figure 1) as an example, it focuses on species with widely accepted medical use and requires the highest level of detail, including compliance with the relevant pharmacopoeia (e.g., the most recent edition of the Chinese, Japanese, Korean, European, Taiwanese pharmacopoeia or the Hong Kong Chinese materia medica standards (HKCMMS). Authors need to decide which one is applicable in their case, and one of these will need to be submitted with (https://ga-online.org/best-practice/).

## Outlook and discussion

Making science open both results in special responsibilities to ascertain reproducibility and offers opportunities to achieve it through higher transparency. With the open-access ConPhyMP tool available, we trust that it will help authors, reviewers, and editors ascertain the reporting level that allows the reproducibility of the research outcomes. Forty years since the first consolidated standards (CONSORT), the ConPhyMP statement is essential for research on extracts as actives. It is a part of ongoing initiatives to ascertain that science remains based on a sound conceptual and methodological basis and, consequently, impactful. We invite all users to contribute to its further development.

## Data Availability

The original contributions presented in the study are included in the article/supplementary material, further inquiries can be directed to the corresponding authors.
